# Ultra-high-resolution photon-counting detector CT in evaluating coronary stent patency: a comparison to invasive coronary angiography

**DOI:** 10.1007/s00330-023-10516-3

**Published:** 2024-01-04

**Authors:** Muhammad Taha Hagar, Martin Soschynski, Ruben Saffar, Moisés Felipe Molina-Fuentes, Jakob Weiss, Alexander Rau, Christopher Schuppert, Philipp Ruile, Sebastian Faby, David Schibilsky, Constantin von zur Muehlen, Christopher L. Schlett, Fabian Bamberg, Tobias Krauss

**Affiliations:** 1 Department of Diagnostic and Interventional Radiology, Medical Center, Faculty of Medicine, University of Freiburg, University of Freiburg, Hugstetter Straße 55, Freiburg im Breisgau, 79106 Germany; 2 Department of Cardiology, Faculty of Medicine, University Hospital Freiburg Heart Centre, Freiburg, Germany, University of Freiburg, Freiburg, Germany; 3 Computed Tomography, Siemens Healthcare GmbH, Forchheim, 91301 Germany; 4 Department of Cardiac and Vascular Surgery, Freiburg University, Freiburg, Germany

**Keywords:** Computed tomography angiography, Stents, Coronary artery disease, Vascular patency

## Abstract

**Objectives:**

To determine the diagnostic accuracy of ultra-high-resolution photon-counting detector CT angiography (UHR PCD-CTA) for evaluating coronary stent patency compared to invasive coronary angiography (ICA).

**Methods:**

Consecutive, clinically referred patients with prior coronary stent implantation were prospectively enrolled between August 2022 and March 2023 and underwent UHR PCD-CTA (collimation, 120 × 0.2 mm). Two radiologists independently analyzed image quality of the in-stent lumen using a 5-point Likert scale, ranging from 1 (“excellent”) to 5 (“non-diagnostic”), and assessed all coronary stents for the presence of in-stent stenosis (≥ 50% lumen narrowing). The diagnostic accuracy of UHR PCD-CTA was determined, with ICA serving as the standard of reference.

**Results:**

A total of 44 coronary stents in 18 participants (mean age, 83 years ± 6 [standard deviation]; 12 women) were included in the analysis. In 3/44 stents, both readers described image quality as non-diagnostic, whereas reader 2 noted a fourth stent to have non-diagnostic image quality. In comparison to ICA, UHR PCD-CTA demonstrated a sensitivity, specificity, and accuracy of 100% (95% CI [confidence interval] 47.8, 100), 92.3% (95% CI 79.1, 98.4), and 93.2% (95% CI 81.3, 98.6) for reader 1 and 100% (95% CI 47.8, 100), 87.2% (95% CI 72.6, 95.7), and 88.6% (95% CI 75.4, 96.2) for reader 2, respectively. Both readers observed a 100% negative predictive value (36/36 stents and 34/34 stents). Stent patency inter-reader agreement was 90.1%, corresponding to a substantial Cohen’s kappa value of 0.72.

**Conclusions:**

UHR PCD-CTA enables non-invasive assessment of coronary stent patency with high image quality and diagnostic accuracy.

**Clinical relevance statement:**

Ultra-high-resolution photon-counting detector CT angiography represents a reliable and non-invasive method for assessing coronary stent patency. Its high negative predictive value makes it a promising alternative over invasive coronary angiography for the rule-out of in-stent stenosis.

**Key Points:**

*• CT-based evaluation of coronary stent patency is limited by stent-induced artifacts and spatial resolution.*

*• Ultra-high-resolution photon-counting detector CT accurately evaluates coronary stent patency compared to invasive coronary angiography.*

*• Photon-counting detector CT represents a promising method for the non-invasive rule-out of in-stent stenosis.*

**Supplementary Information:**

The online version contains supplementary material available at 10.1007/s00330-023-10516-3.

## Introduction

Recent guidelines recommend coronary CT angiography (CTA) as the modality of choice for evaluating coronary arteries in patients with stable chest pain and low to intermediate risk of coronary artery disease [1, 2]. However, coronary CTA is generally less recommended in patients with prior coronary stent implantation due to the higher risk profile of these patients [3]. Furthermore, stent-induced blooming and beam-hardening artifacts compromise the diagnostic value of cardiac CTA [4–7]. Stent-induced blooming has been mainly attributed to partial volume averaging, which is influenced by spatial resolution and detector cell size [8, 9], leading to overestimation of stenosis [10]. However, non-invasive assessment of coronary stent patency is desirable to avoid unnecessary invasive coronary angiography (ICA).

Novel photon-counting detector CT (PCD-CT) technology might overcome these limitations, providing higher spatial resolution as the direct conversion process of absorbed X-ray photons requires no additional septa between detector pixels [11]. Moreover, electrocardiogram (ECG)–synchronized ultra-high-resolution photon-counting detector CT angiography (UHR PCD-CTA) at a maximum in-plane resolution of 0.11 mm is available [12].

Phantom studies provided promising results of PCD-CT for stent assessment with reduced artifacts in UHR imaging, especially when a sharp vascular convolution kernel is applied [13, 14].

Initial in-human results point to an improved image quality of PCD-CT compared to energy-integrating detector CT in patients referred for coronary stent imaging [15]. Especially, UHR PCD-CTA displayed excellent in vivo stent lumen visualization [16].

However, there is limited evidence on the diagnostic performance of PCD-CTA for coronary stent evaluation compared to a reference standard. Therefore, our study aimed to investigate the accuracy of UHR PCD-CTA in the assessment of coronary stent patency compared to ICA as the standard of reference.

## Materials and methods

### Ethics statement

The study was performed in accordance with the ethical principles of the Declaration of Helsinki. Approval of our study protocol was granted on 09/21/2021 (No. 21-2469) by the institutional review board of the University of Freiburg Medical Center, and all participants provided informed written consent prior to the inclusion.

### Patients

We prospectively enrolled consecutive patients who underwent UHR PCD-CTA as part of their pre-transcatheter aortic valve implantation (TAVI)-CT workup routine comprising diagnostic ICA and TAVI-CT at the University of Freiburg Medical Center between August 2022 and March 2023. Additional inclusion criteria included previous coronary stent implantation and diagnostic ICA within 8 weeks of PCD-CTA. Exclusion criteria were as follows: patients who presented a contraindication to contrast-enhanced CT. Some study subjects have been previously reported [17]; however, the current study has a different scope, focusing on stented patients, and includes more stented participants.

### Invasive coronary angiography

ICA was performed by two expert board-certified interventional cardiologists: a radial approach was used to insert a 6-F sheath (Glidesheath Slender, Terumo) and a 6-F catheter (usually Judkins right 4, Judkins left 3.5). Diagnostic angiography was performed with a standard sequence of six projections (four views for the left and two views for the right coronary system) and an average contrast volume of 50 mL. ICA images were assessed by an independent board-certified interventional cardiologist (10 years of experience) blinded to the results of CTA. Significant in-stent stenosis was defined as 50% or greater diameter stenosis within the stented lumen [18]. Disagreements between the board-certified ICA reader and a preceding ICA report were resolved by consultation of a second expert board-certified cardiologist (C.V.Z.M., 15 years of experience) who independently read the ICA images and subsequently made a final adjudication on in-stent stenosis severity, which served as the standard of reference.

### CT angiography

All participants were scanned on a clinical, dual-source PCD-CT scanner (NAEOTOM Alpha, software version syngo CT VA-50, Siemens Healthineers). In alignment with established TAVI-CT imaging guidelines, neither nitroglycerin nor beta-blockers were administered on the day of the examination [19]. Detailed technical and scan parameters for UHR PCD-CTA are provided in Supplementary Table 1. CTA was initiated via bolus tracking 10 s after attenuation exceeded 130 Hounsfield Units in a region of interest (ROI) placed in the aortic root. A total of 70 mL of contrast media, Ultravist 370 (Bayer Healthcare), followed by a solution of 40 mL isotonic saline and 30 mL contrast medium, was administered using a dual-syringe power injector at a flow rate of 5.0 mL/s each. Automatically determined best-systolic and best-diastolic phases and multiphase data were reconstructed using a sharp vascular convolution kernel (Bv-60 with quantum iterative reconstruction at level 3). The matrix size was set at 1024^2^ pixels. The field of view was restricted to the heart at 180 × 180 mm and a slice thickness of 0.2 mm with an increment of 0.1 mm was employed.

### Assessment of CT data

Two radiologists with 3 years (R.S.) and 4 years (M.T.H.) of experience in cardiovascular CT imaging assessed all images independently on a dedicated workstation (syngo.via, version VB60, Siemens Healthineers) while blinded to clinical data, including ICA results. All patients’ radiation dose parameters, specifically the dose length product and the dose volume CT index, were extracted from the CT reports.

#### Subjective image quality

**Fig. 1:**
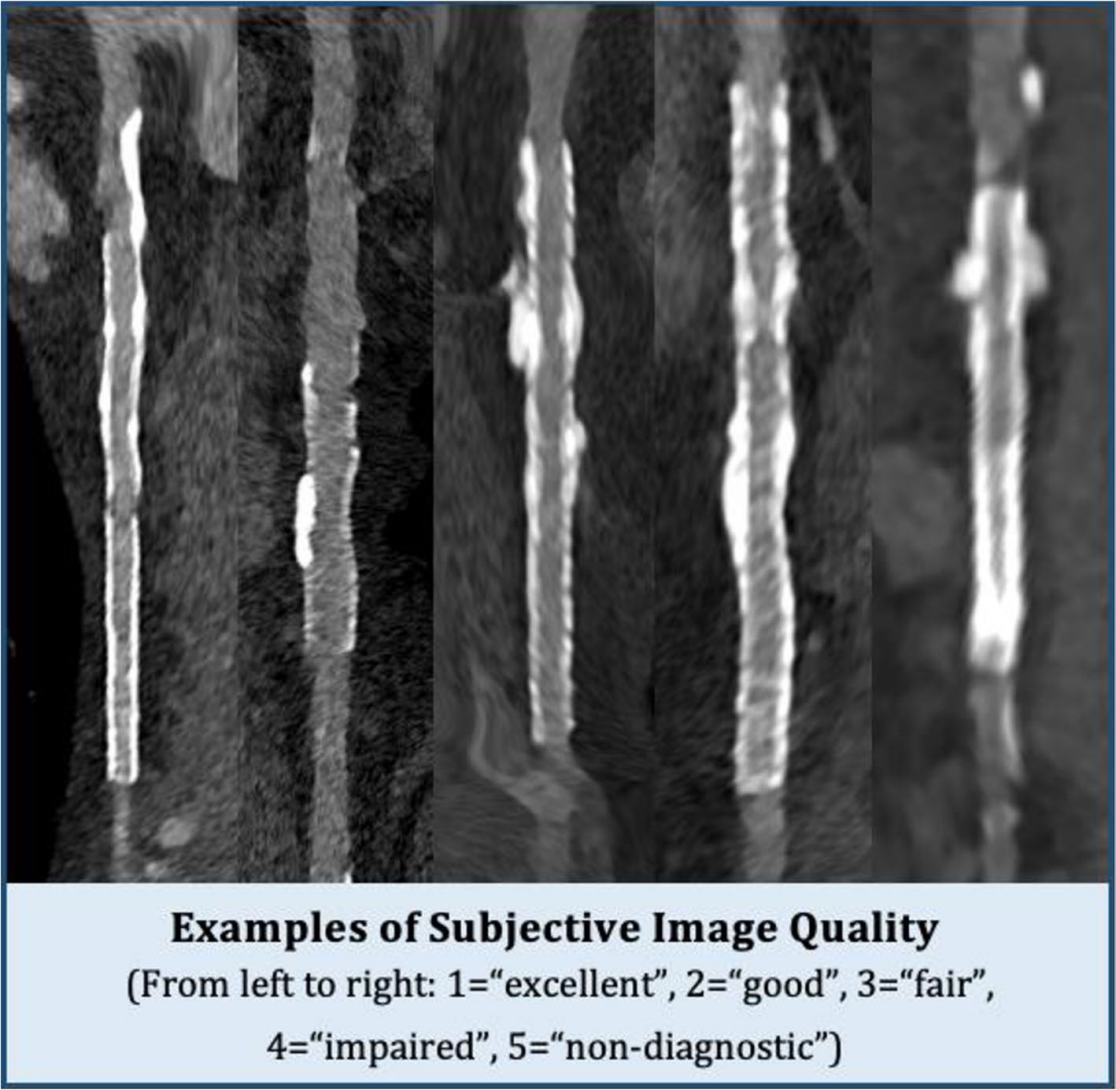
Multiplanar reformation of stented coronary vessels for subjective image quality assessment. The figure demonstrates examples across the 5-point Likert scale used for evaluating image quality. Two readers independently assessed each stent depicted and arrived at congruent subjective image quality scores

The *subjective image* quality of the in-stent lumen was individually assessed for each stent using a 5-point Likert scale (Fig. 1). A score of 1 denoted “excellent” image quality, signifying clear and artifact-free images with perfect lumen visibility. A score of 2 signified “good” image quality, representing images with minimal artifacts and clear lumen visualization. A score of 3 suggested “fair” image quality, marking adequate images with some detectable artifacts. A score of 4, termed “impaired,” indicated compromised image quality due to noticeable artifacts but still sufficient for diagnosis, and lastly, a score of 5, labeled “non-diagnostic,” corresponded to low image quality with a high presence of artifacts, unsuitable for diagnosis of stent patency.

#### Objective image quality: stent-induced blooming

Both readers independently measured coronary stent length as well as internal and external stent diameters on multiplanar reformatted CT images, focusing on the axial slice that best represents the stent geometry for each case. When two stents were connected, each stent was measured independently, and an averaged metric was computed for the purpose of assessing stent-induced blooming. There was no limitation on the maximum number of stents that could be assessed per vessel. The estimated stent-induced blooming was assessed according to the methodology described by Boccalini et al [20]:

$$\mathrm{Blooming}\;\mathrm{estimate}\;\lbrack\%\rbrack=\frac{\mathrm{stent}\;{\mathrm{diameter}}_\text{external}\;\lbrack\text{mm}\rbrack-\mathrm{stent}\;{\mathrm{diameter}}_\text{internal}\;\lbrack\text{mm}\rbrack}{{\mathrm{stendiameter}}_\text{external}\lbrack\text{mm}\rbrack}\;\times\;100$$

#### Objective image quality: effect on the in-stent lumen attenuation

A total of three manually drawn ROIs were used: The first, designated as (ROIin_stent), was drawn to be as large as possible to cover the in-stent lumen while carefully avoiding vessel walls and stent struts and had a minimum area of 4 mm^2^. Two other ROIs of the same size were placed in the coronary artery lumen—one directly proximal to the stent (ROIprox) and the other distal to the stent (ROIdist), both within a maximum distance of 10 mm from the stent edges. When calcification was present at one end, an ROI was placed as close as possible to the calcified area while avoiding the calcification itself. From these ROIs, the average HU was read. Stent struts, vessel walls, and calcified or non-calcified plaques were carefully avoided. The following equation served as a metric to quantify the stent-induced effect on the in-stent lumen attenuation (Figure S1) [20]:

$$\Delta {\text{HUin}}\_{\text{stent}}={\text{ROIin}}\_\mathrm{stent }[{\text{HU}}]- \frac{\mathrm{ROIprox }\left[{\text{HU}}\right]+\mathrm{ROIdist }[{\text{HU}}]}{2}$$

#### Evaluation of stent patency

In-stent stenosis quantification was performed independently by both readers. Significant in-stent stenosis was defined as diameter stenosis of 50% or greater. In the absence of in-stent stenosis, a stent was deemed to be patent. Following an intention-to-diagnose approach, all segments of the in-stent lumen with non-diagnostic image quality were rated as potentially significant in-stent stenosis.

### Statistics

IBM SPSS Statistics for Macintosh (version 28.0) and R (version 4.3.0, https://www.R-project.org/) were used for statistical analysis. A one-sample Shapiro-Wilk test was applied to check for the assumption of normal distribution. Quantitative variables were expressed as number (percentage), mean ± standard deviation, or median [interquartile range], as appropriate. The overall subjective image quality given by both readers was compared using the Wilcoxon signed-rank test, whereas the McNemar test was used to compare the individual Likert values between readers. Independent measurements of stent diameters and estimates of blooming were compared using paired *t*-test. The Wilcoxon signed-rank test was used to test for the differences between both observers in terms of attenuation measurements in the stent lumen and adjacent coronary vessel. Interobserver agreement regarding the evaluation of stent patency was expressed in Cohen’s kappa (*κ*) value and interpreted as follows: ≤ 0.20 none, 0.21–0.40 fair, 0.41–0.60 as moderate, 0.61–0.81 as substantial, and ≥ 0.82 as very strong agreement [21]. To evaluate the diagnostic performance of UHR PCD-CTA for the assessment of stent patency, we compared the results of the CTA reading to the ICA results, serving as the reference standard, and further calculated sensitivity, specificity, positive predictive value (PPV), negative predictive value (NPV), and diagnostic accuracy. To account for the potential clustering of diagnostic performance values of multiple stents per patient, we calculated the intracluster correlation coefficients (ICC) [22]. A 95% confidence interval range (CI) was calculated and expressed for the results of all diagnostic accuracy tests. A two-tailed *p*-value of < 0.05 was considered statistically significant.

## Results

### Patients’ characteristics

**Fig. 2:**
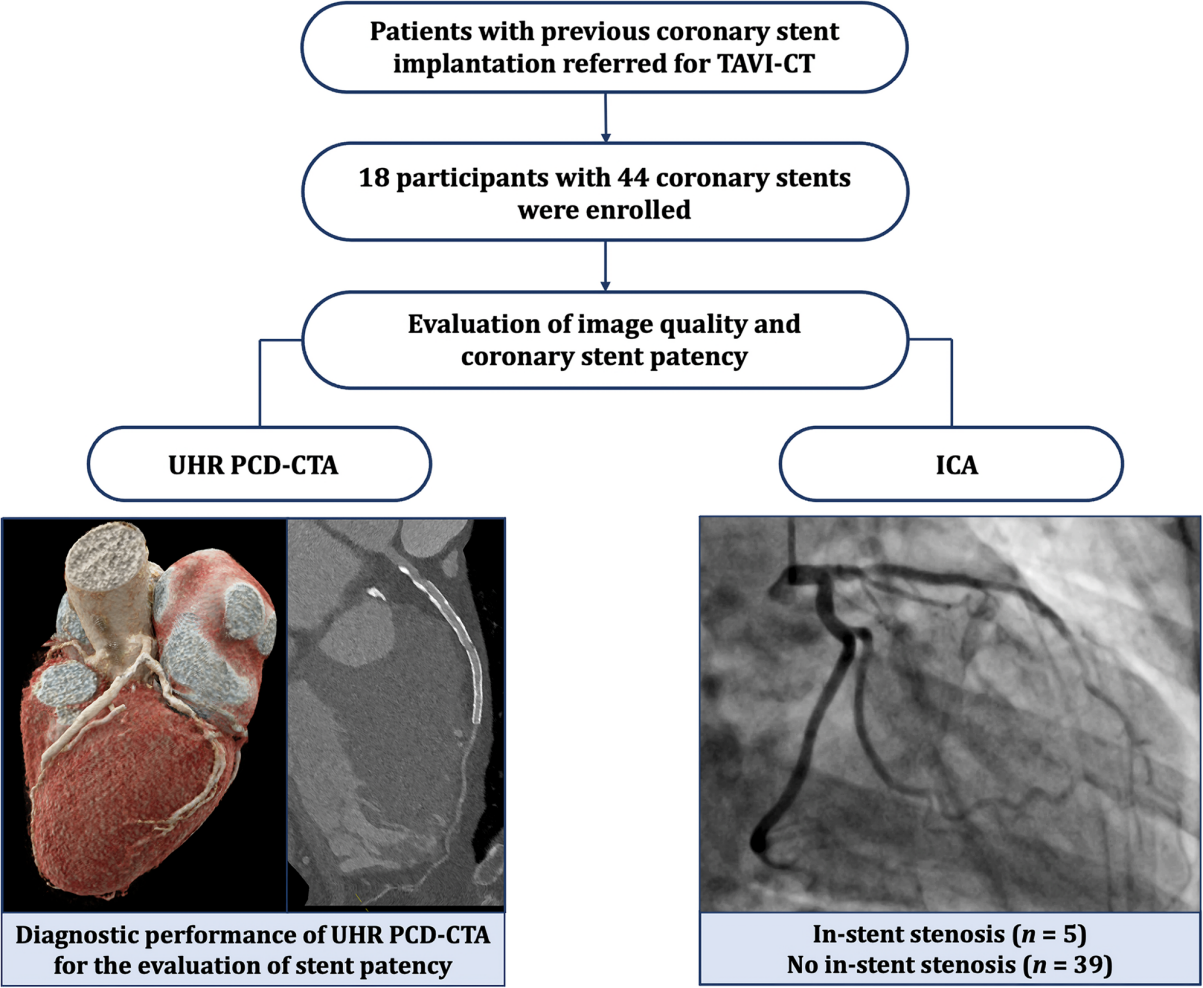
Participants’ flowchart. The diagnostic performance of UHR PCD-CTA for evaluating stent patency in 18 patients with a total of 44 stents was determined in comparison to ICA, serving as the standard of reference. Diameter stenosis of 50% or greater was defined as relevant in-stent stenosis. Abbreviations: TAVI transcatheter aortic valve implantation, UHR PCD-CTA ultra-high-resolution photon-counting detector computed tomography angiography, ICA invasive coronary angiography

A total of 18 patients (mean age, 83 years ± 6 [standard deviation]; 12 women) with a total of 44 coronary stents were included. No patients were excluded. Information on the flowchart is given in Fig. 2. Patients’ characteristics, including information on radiation dose, and information on the stents are provided in Table 1.

**Table 1:** Participants’ characteristics and stent information

Characteristics	Values
*Participant characteristics*	
Number of participants	18 (100%)
Age (years)	82.6 ± 5.9
Sex	
Females	12 (66.7%)
Males	6 (33.3%)
Weight (kg)	74.4 ± 11.4
BMI (kg/m^2^)	25.7 ± 3.0
*Cardiovascular risk factors*	
History of myocardial ischemia	3 (16.7%)
History of coronary stenting	18 (100%)
Arterial hypertension	13 (72.2%)
Diabetes mellitus	7 (38.9%)
Hyperlipidemia	15 (83.3%)
Chronic kidney disease (eGFR < 45 mL/min/1.73 m^2^)	7 (38.9%)
Smoking	6 (33.3%)
Tube voltage	
120 kV	8 (44.4%)
140 kV	10 (55.6%)
Radiation dose	
CTDIvol (mGy)	70.2 [64.9–80.9]
DLP (mGy*cm)	994 [878–1086]
*Stent information*	
Number of stents	44 (100%)
Location	
LM	2 (4.5%)
LAD	17 (36.4%)
LCx	14 (29.5%)
RCA	11 (22.7%)
Diameter	
> 3 mm	31 (70.4%)
≤ 3 mm	13 (29.6%)
Length	
> 20 mm	23 (52.3%)
≤ 20 mm	21 (47.7%)

Data are presented as mean ± standard deviation, number and frequencies in parentheses, and as median and interquartile range in square brackets

Abbreviations: *BMI* body mass index, *eGFR* estimated glomerular filtration rate, *CTDIvol* volume computed tomography dose index, *DLP* dose length product, *LM* left main artery, *LAD* left anterior descending artery, *LCx* left circumflex artery, *RCA* right coronary artery

### Evaluation of subjective and objective image quality

**Fig. 3:**
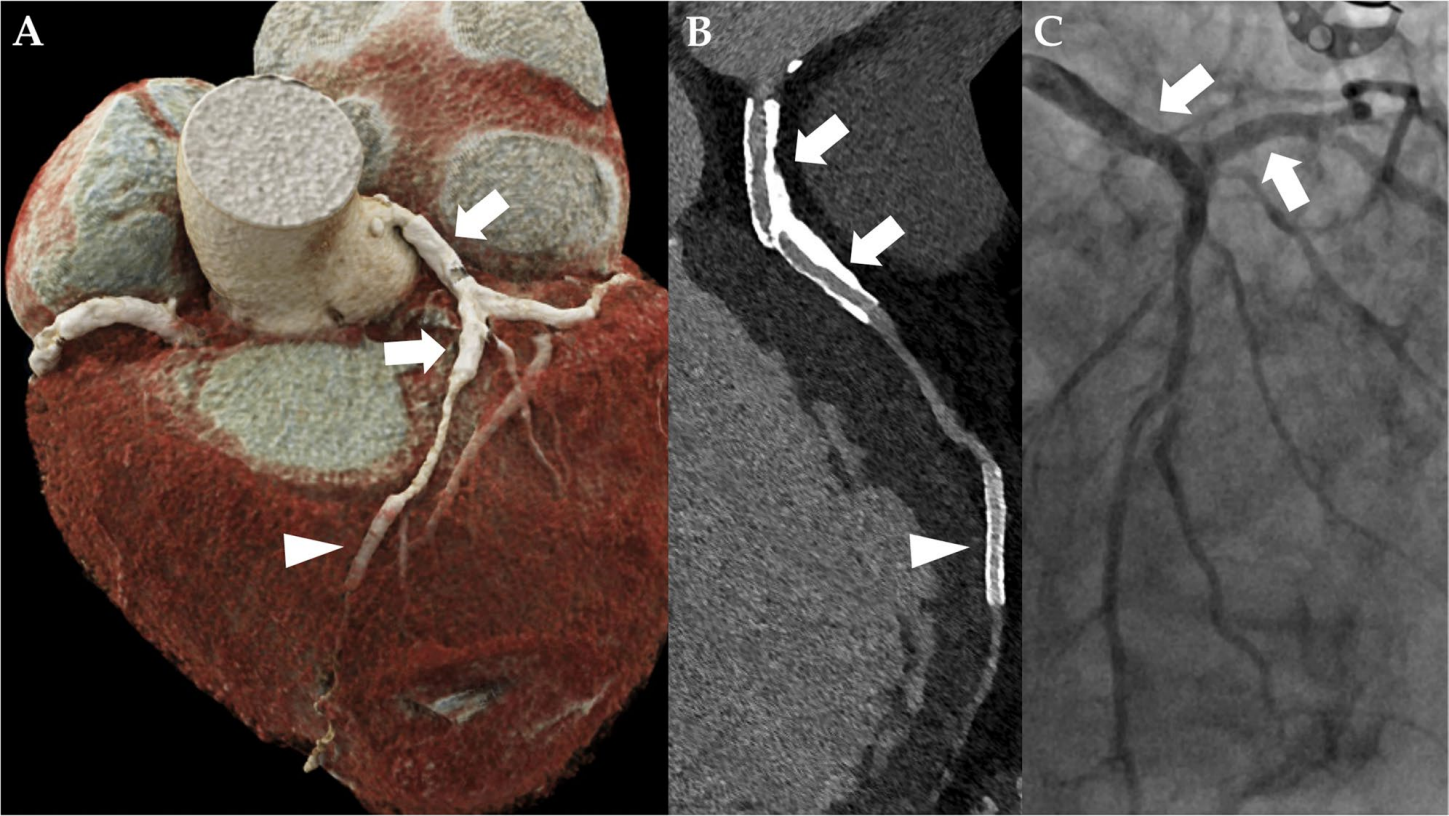
Ultra-high-resolution photon-counting detector CT angiography (PCD-CTA) of the heart of an 89-year-old male patient with severe aortic valve stenosis and a history of coronary stent implantation. **A** Three-dimensional cinematic rendering of the heart. There is a kissing stent in the left main artery, proximal left circumflex artery, and proximal left anterior descending artery (LAD) (white arrow). Note two additional stents in the distal LAD (arrowhead) and in the proximal right coronary artery. **B** Curved multiplanar reformation of the LAD. In-stent stenosis could be confidently excluded in UHR PCD-CTA by both readers. The proximal kissing stent was valued as excellent (Likert 1) by both readers, and the distal LAD stent was valued as “impaired”—Likert 4 by one reader—and as “moderate”—Likert 3 by another. **C** Invasive coronary angiography of the same patient with a 30° right anterior oblique view, verifying stent patency

**Fig. 4:**
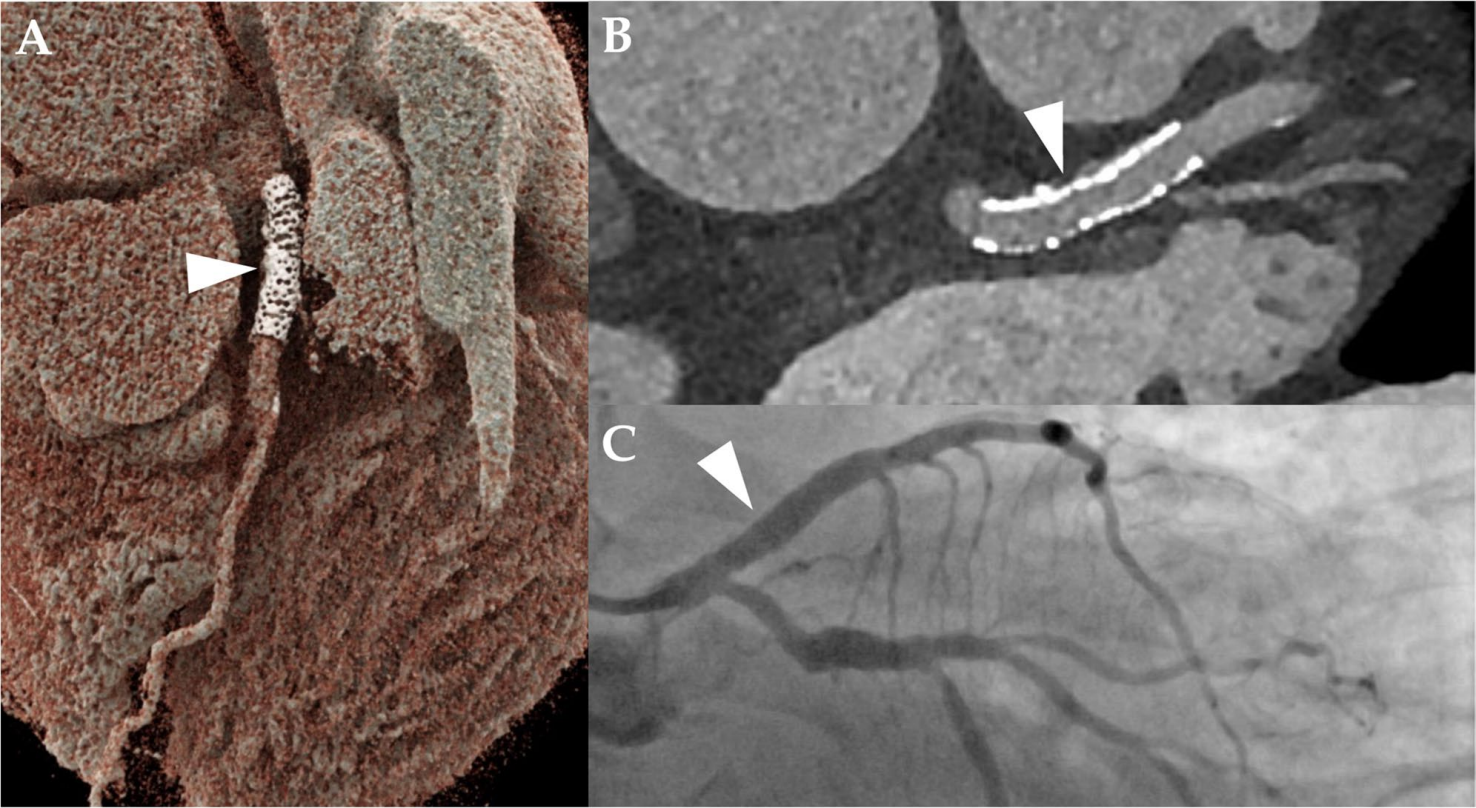
Ultra-high-resolution photon-counting detector CT angiography (PCD-CTA) of the heart of a 69-year-old male patient with severe aortic valve stenosis and a history of coronary stent implantation in the distal left main and proximal left anterior descending artery (LAD) (arrowhead in **A**, **B**, and **C**). **A** Three-dimensional cinematic rendering of the heart, showing the stent in the LAD. **B** Curved multiplanar reformation showing the absence of in-stent stenosis. The stent was rated as “excellent”—Likert 1 by one reader—and as “good”—Likert 2 by the other reader. **C** Invasive coronary angiography at caudal 30° view, verifying stent patency

**Fig. 5:**
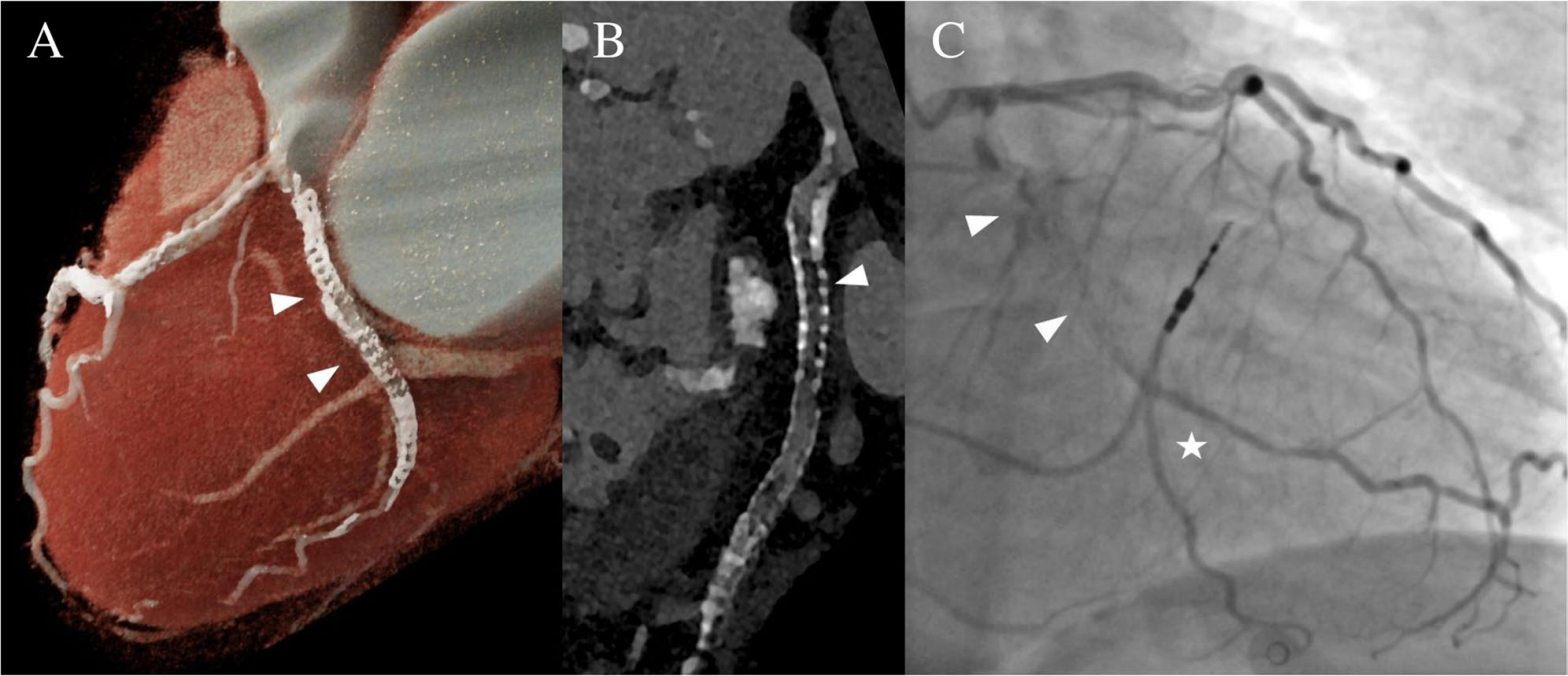
Ultra-high-resolution photon-counting detector CT angiography in a 72-year-old female with severe aortic valve stenosis and a history of coronary stenting. **A** Three-dimensional cinematic reformation illustrating the stent in the left circumflex coronary artery (arrowheads). **B** Curved multiplanar reformation of the proximal left circumflex coronary artery reveals an intraluminal hypoattenuation (arrowhead), suggesting severe stenosis or complete obstruction of the in-stent lumen. **C** Invasive angiography (RAO 20°, CAU 10°) corroborates the obstruction of the in-stent lumen (arrowheads). Notably, the vessel’s distal part is perfused retrogradely through collateral channels (star)

In 3/44 stents, both readers described image quality as non-diagnostic, whereas reader 2 noted a fourth stent to have non-diagnostic image quality. The overall subjective image quality of the stent lumen was rated as good to excellent by both readers (median score, 1 [IQR, 1–2.3] vs. median score, 2 [IQR, 1–2.3]; *p* = 0.38). An overall blooming estimate of 37.9 ± 9.6% (reader 1) and 40.1 ± 8.9% (reader 2) was observed (*p* = 0.03). Stents noticeably affected CT attenuation within the vessel lumen, as a median ∆HU_in-stent_ of + 77 HU [IQR, 36–106], according to reader 1, and a median ∆HU_in-stent_ of + 18 HU [IQR − 1 to 100], according to reader 2, were registered (*p* = 0.02). Detailed metrics of subjective and objective image quality are provided in Table 2. Imaging examples for patent stents are given in Figs. 3, 4 and 5 provides an example demonstrating in-stent stenosis.

**Table 2:** Subjective and objective image quality

	Reader 1	Reader 2	*p*-value
Subjective image quality			
Excellent (Likert 1)	23 (52.3%)	16 (36.4%)	0.92
Good (Likert 2)	10 (22.7%)	17 (38.6%)	0.14
Fair (Likert 3)	4 (9.1%)	6 (13.6%)	0.75
Poor (Likert 4)	4 (9.1%)	1 (2.3%)	0.38
Non-diagnostic (Likert 5)	3 (6.8%)	4 (9.1%)	> .99
Overall image quality	1 [1–2.3]	2 [1–2.3]	0.38
Objective image quality			
* Stent blooming*			
Measured outer diameter (mm)	3.7 ± 0.7	3.8 ± 0.7	0.02
Measured inner diameter (mm)	2.3 ± 0.7	2.3 ± 0.6	0.52
Blooming estimate (%)	37.9 ± 9.6	40.1 ± 8.9	0.03
* Stent lumen*			
Attenuation proximal lumen (HU)	480 [409–499]	468 [396–523]	0.87
Attenuation distal lumen (HU)	455 [378–511]	453 [405–519]	0.13
Attenuation in-stent lumen (HU)	518 [466–601]	496 [425–577]	0.18
∆HU in-stent lumen (HU)	77 [36–106]	18 [− 1 to 100]	0.02

Data are presented as number and frequencies in parenthesis, mean ± standard deviation, and median and interquartile range in square brackets

Abbreviations: *mm* millimeter, *HU* average Hounsfield Units

### Diagnostic performance of PCD-CTA for the evaluation of coronary stent patency

ICA identified five in-stent stenosis, resulting in a prevalence of 11.4% (5/44). Correspondingly, 88.6% (39/44) of stents were found to be patent. Both readers correctly recognized all in-stent stenosis. Following an intention-to-diagnose approach, we observed three false-positive findings for the first reader and four false-positive findings for the second reader. Sensitivity, specificity, and accuracy for in-stent stenosis of 50% or greater were 100% (95% CI 47.8, 100), 92.3% (95% CI 79.1, 98.4), and 93.2% (95% CI 81.3, 98.6) according to reader 1, and 100% (95% CI 47.8, 100), 87.2% (95% CI 72.6, 95.7), and 88.6% (95% CI 75.4, 96.2) according to reader 2, as shown in Table 3. As the ICCs for sensitivity, specificity, accuracy, PPV, and NPV were small for each of the readers (ICC ≤ 0.21), the cluster effect on mean estimates and standard errors of these diagnostic performance parameters was deemed negligible. PPV for both readers was 62.5% (95% CI 36, 83) and 50% (95% CI 30.6, 69.4), respectively. Both readers observed an NPV of 100% (36/36 stents for reader 1 and 34/34 stents for reader 2). The inter-reader agreement for the assessment of stent patency was 90.1%, corresponding to a substantial Cohen’s *κ* value of 0.72.

**Table 3:** Diagnostic accuracy of UHR PCD-CTA for the presence of in-stent stenosis ≥ 50%

	Reader 1	Reader 2
*Per stent (n = 44)*		
Sensitivity (%)	100 (47.8, 100) [5/5]	100 (47.8, 100) [5/5]
Specificity (%)	92.3 (79.1, 98.4) [36/39]	87.2 (72.6, 95.7) [34/39]
PPV (%)	62.5 (36, 83) [5/8]	50 (30.6, 69.4) [5/10]
NPV (%)	100 (NA) [36/36]	100 (NA) [34/34]
Accuracy (%)	93.2 (81.3, 98.6) [41/44]	88.6 (75.4, 96.2) [39/44]
*Per participant (n = 18)*		
Sensitivity (%)	100 (29.2, 100) [3/3]	100 (29.4, 100) [3/3]
Specificity (%)	80 (51.9, 95.7) [12/15]	66.7 (38.4, 88.2) [10/15]
PPV (%)	50 (26.7, 73.3) [3/6]	37.5 (22.7, 55.1) [3/8]
NPV (%)	100 (NA) [12/12]	100 (NA) [10/10]
Accuracy (%)	83.3 (58.6, 96.4) [15/18]	72.2 (46.5, 90.3) [13/18]

Data in parenthesis are 95% confidence interval range. Data in square brackets are raw data

Abbreviations: *UHR PCD-CTA* ultra-high-resolution photon-counting detector CT angiography, *ICA* invasive coronary angiography, *PPV* positive predictive value, *NPV* negative predictive value, *NA* not applicable

## Discussion

In this study, we explored the clinical utility of UHR PCD-CTA in assessing coronary stent patency, focusing on both image quality and diagnostic accuracy. The principal findings can be summarized as follows: (1) A majority of stents were rated with good to excellent image quality in subjective assessments, while objective assessments indicated limited artifact interference. (2) UHR PCD-CTA demonstrated a diagnostic accuracy exceeding 88% compared to the established reference standard of ICA. (3) Both readers observed an NPV of 100%, and (4) the inter-reader agreement was found to be substantial, with a Cohen κ value of 0.72. These findings indicate that novel UHR PCD-CTA is a reliably non-invasive tool for assessing coronary stent patency.

We observed that combining UHR PCD-CTA for TAVI planning and coronary in-stent lumen assessment is feasible with an assessability above 90%. This superseded a study on a third-generation dual-source energy-integrating CT scanner for TAVI candidates that showed an assessability of coronary stents of 79.6% [23]. Our study’s non-diagnostic coronary stent rate of less than 10% is in line with a meta-analysis on multi-detector CT, which reported an overall feasibility of 90.4% [24]. However, none of their analyzed studies included TAVI candidates, a cohort presenting unique challenges for coronary CTA. Specifically, nitroglycerine and beta-blockers are contraindicated in these patients due to the high risk of sudden blood pressure drop associated with severe aortic valve stenosis, as indicated in the 2019 Society of Cardiovascular Computed Tomography guidelines on CT imaging in the context of TAVI [19].

In a recent study on a prototype PCD-CT scanner with high-resolution scan mode and a maximum in-plane resolution of 0.21 mm, a blooming estimate of 53.4% was observed [15]. In our study, less blooming was observed, which we attribute to the reduced spatial resolution of 0.11 mm of the dual-source PCD-CT and the employment of a sharp vascular convolution kernel. Besides spatial resolution, many other factors influence the amount of blooming, such as iterative reconstruction strength, kernel, and slice thickness [25]. A novel phantom study found that employing a sharp vascular convolution kernel on UHR PCD-CTA improves lumen visibility [13]. In a study on 20 patients, the investigators observed that the use of a slice thickness of 0.2 mm and quantum iterative reconstruction, combined with a sharp vascular kernel, improved the visualization of lipid-rich components and reduced blooming of calcified plaques in coronary UHR PCD-CTA [26]. Our results are consistent to these studies, as we observe excellent image quality and low blooming on UHR PCD-CTA applying comparable image reconstruction parameters (0.2 mm slice thickness, sharp vascular convolution kernel Bv60, and quantum iterative reconstruction 3). Potentially, blooming could have been further reduced in our study if we had applied quantum iterative reconstruction at the highest possible strength. Using an established method to quantify the influence of the stent on the in-stent lumen attenuation [15], we observed a statistically significant increase in the Hounsfield Units (HU) within the stent lumen. Specifically, reader 1 observed an increase of + 77 HU, while reader 2 noted an increase of + 18 HU, with a *p*-value of 0.02, indicating a significant difference between the two sets of readings. This trend was also found in the study by Boccalini et al, although they reported slightly higher values of in-stent lumen attenuation [15]. The differences in our findings compared to Boccalini et al and between our own readers suggest that the method, while consistent in demonstrating increased attenuation, may lack reliability.

The overall diagnostic accuracy in our study was excellent, with excellent sensitivity and negative predictive value (100% each). Yet, non-assessable stents constitute a limitation and explain low PPV. Our results regarding limited PPV are consistent to a study investigating the diagnostic performance of coronary stent evaluation of a whole-organ energy-integrating CT scanner in a less challenging patient collective, leading to a PPV of 60% in 100 consecutive patients [27]. Due to CTA’s limited PPV for in-stent imaging, careful patient selection is mandatory, such as a positive stress test or with clinical suspicion of in-stent stenosis, to prevent unnecessary invasive follow-up procedures caused by non-assessable stents [28].

To date, the full potential of clinical dual-source PCD-CTA in assessing coronary stents is not yet exploited, as the current software version only allows for the acquisition of spectral images (Quantum Plus mode) or UHR mode. Image quality and, consequently, diagnostic performance might be further improved on PCD-CTA by combining spectral information and the UHR scan mode—enabling reconstructions with iodine maps or a calcium and metal removal algorithm (PURE Lumen), which could further enhance in-stent imaging [29].

Our study has some limitations: first, the limited number of participants requires additional confirmatory research to make a statement about generalizability. Moreover, due to the limited sample size, no meaningful subgroup analysis could be performed (i.e., assessing the diagnostic accuracy of UHR PCD-CTA in small stents). Second, we did not compare the diagnostic performance of UHR PCD-CTA to energy-integrating detector CTA or spectral PCD-CTA. Third, we lacked clinical data on many patients regarding stent type, stent material composition, and date of implantation due to external referral. Our study specifically enrolled high-risk patients who were candidates for TAVI. Consequently, the diagnostic performance findings could be subject to selection bias, limiting their applicability to a broader population. Further, when interpreting the results of our diagnostic tests, it should be taken into account that the established gold standard for in-stent plaque quantification is optical coherence tomography and intravascular ultrasound [30]—we compared our CT reading results to ICA, which represents the standard of reference in clinical routine. Lastly, as this was the subject of previous studies, we did not perform additional analysis on the effect of different kernels on image quality.

Future studies with the inclusion of patients with suspected in-stent stenosis and a focus on patient-related endpoints should be performed to further evaluate the contributory value of PCD-CTA to clinical decision-making and patient care.

## Conclusions

UHR PCD-CTA enables non-invasive assessment of coronary stent patency with high image quality and diagnostic accuracy. Its high negative predictive value shows great promise in non-invasively ruling out in-stent stenosis as an alternative to invasive coronary angiography.

### Supplementary Information

Below is the link to the electronic supplementary material.

Supplementary file1 (PDF 292 KB)

## Abbreviations

CI: Confidence interval
CTA: Computed tomography angiography
ECG: Electrocardiogram
ICA: Invasive coronary angiography
NPV: Negative predictive value
PCD-CT: Photon-counting detector computed tomography
PPV: Positive predictive value
ROI: Region of interest
UHR PCD-CTA: Ultra-high-resolution photon-counting detector CT angiography

## Declarations

### Guarantor

The scientific guarantor of this publication is Dr. Tobias Krauss.

### Statistics and biometry

One of the authors, Martin Soschynski, has significant statistical expertise.

### Study subjects or cohorts overlap

Some study subjects have been previously reported in Hagar, Muhammad Taha, et al. “Accuracy of ultrahigh-resolution photon-counting CT for detecting coronary artery disease in a high-risk population.” Radiology 307.5 (2023): e223305. The current study includes more stented patients and has another scope, focusing specifically on stent imaging.

### Methodology

• prospective

• observational

• performed at one institution
