# Supplementary material for: Ultra-high-resolution photon-counting detector CT in evaluating coronary stent patency: a comparison to invasive coronary angiography
Source: Eur Radiol. 2024 Jan 4;34(7):4273–83. doi: 10.1007/s00330-023-10516-3 (PMC11213791; doi:10.1007/s00330-023-10516-3)
Supplement: Supplementary file 1 — Supplementary file1 (PDF 292 KB) [file 330_2023_10516_MOESM1_ESM.pdf]

Ultra-high-resolution photon-counting detector CT in evaluating coronary stent patency: a comparison to invasive coronary angiography

Electronic Supplementary Material (ESM)

Eur Radiol (2023) Hagar MT, Soschynski M, Saffar R et al

Supplementary Table 1: Technical parameters for ultra-high-resolution photon-counting detector CT-angiography

**Technical parameters**	**Values**
Scan mode	ECG-synchronized retrospective spiral CTA
z-coverage	120 x 0.2 mm
Spatial resolution	0.11 mm in-plane
Gantry rotation time	250 ms
Pitch	Depending on the patient's heart rate, preset at 0.2
Spectral information	Not available at ultra-high-resolution scan
Dose modulation	ECG-pulsing set at 15 – 80 % of the R–R interval
Tube voltage	Automatically determined (Care kV), either 120kV or 140 kV
Tube current	Automatic tube current modulation (CareDose mAs)

Abbreviations: *ECG* electrocardiogram, *CTA* computed tomography angiography, *R–R interval* time elapsed between two consecutive R waves of a patient's electrocardiogram.

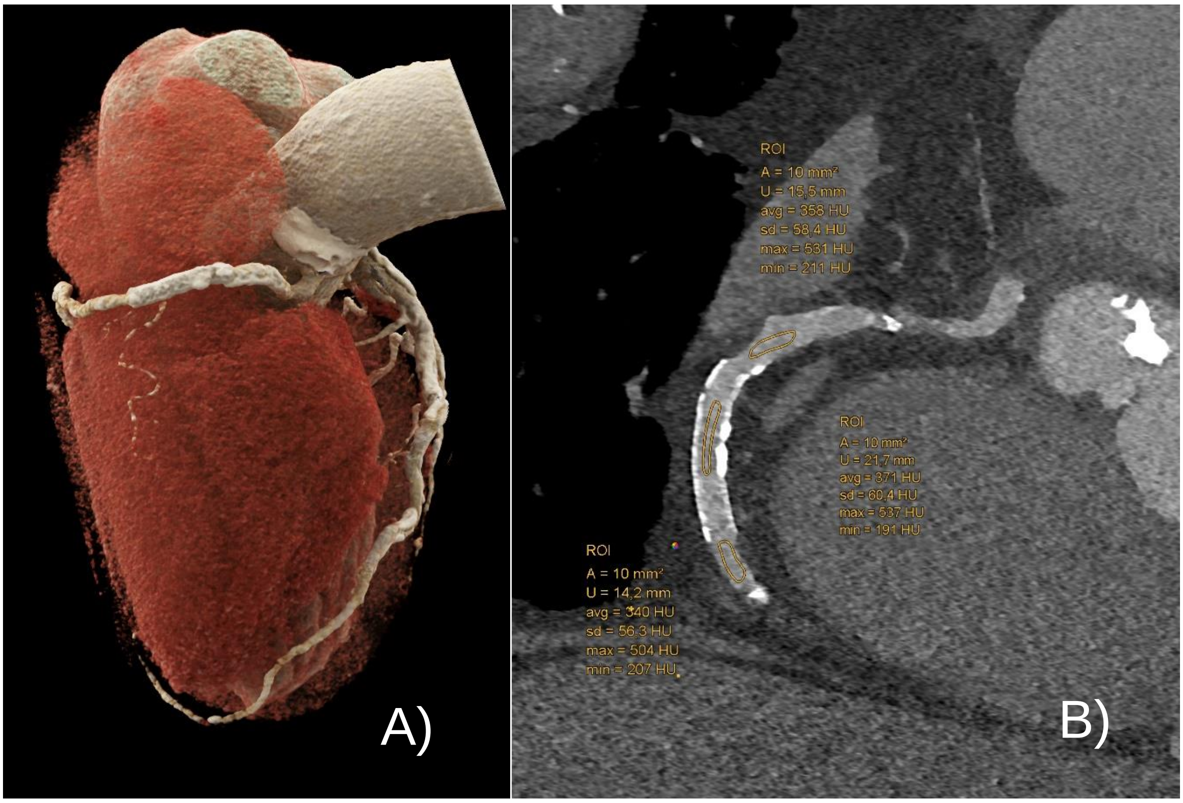

Supplementary Figure 1. Ultra-high-resolution photon-counting CT-angiography of an 85-year-old patient with previous stent implantation in the right coronary artery. A: Three-dimensional cinematic rendering showing the stent in segment 2 of the right coronary artery (RCA). B: Multiplanar reformation of the right coronary artery.

To quantify the stent-induced effect on the in-stent lumen attenuation (Δ*HUin_stent*), manually drawn regions of interest (ROI) are placed in the stent lumen (ROI_{in_stent}) and ROIs of the same size (here: $10mm^2$) in the proximal (ROI_{prox}) and distal (ROI_{dist}) adjacent coronary vessel. The average Hounsfield Units (HU) are noted and Δ*HUin_stent is calculated as follows:*

$$\Delta\mathrm{HUin\_stent} = \mathrm{ROIin\_stent\ HU} - \frac{\mathrm{ROIprox\ HU+ROIdist\ HU}}{2} \quad => \quad 371\ \mathrm{HU} - \frac{(358\ \mathrm{HU} + 340\ \mathrm{HU}\ )}{2} = +22\ \mathrm{HU}.$$
